# Viscoelastic Properties and Enzymatic Degradation of Crosslinked Hyaluronic Acid for Deep Dermal Filler Use

**DOI:** 10.3390/gels11090754

**Published:** 2025-09-18

**Authors:** Alejandro Melero, Jon Andrade del Olmo, Nagore Martínez de Cestafe, Claudia Goenaga Ibeas, Miguel Ucelay López de Heredia, Jon Kepa Izaguirre, José María Alonso, Raúl Pérez González

**Affiliations:** i+Med S. Coop., Alava Technology Park, C/Hermanos Elhuyar 6, 01510 Vitoria-Gasteiz, Spain

**Keywords:** crosslinked hyaluronic acid, dermal fillers, aesthetic medicine, facial volume restoration

## Abstract

Crosslinked hyaluronic acid dermal fillers are widely used for non-permanent aesthetic enhancement, offering safe and effective solutions for facial volume restoration. Specific formulations are designed for targeted facial regions, with highly crosslinked hydrogels often recommended for volumizing areas such as the jawline, chin, and cheeks due to their structural properties. While elasticity and viscosity are commonly evaluated, broader comparative analyses remain limited. In this study, we assess five commercially available HA-based fillers with similar HA concentrations, all optimised for volume enhancement. Alongside widely used reference products, we evaluate BtHCROSS 2%^®^, a novel formulation not previously compared to established fillers. We examined the degree of chemical modification, mechanical viscoelastic behaviour, susceptibility to enzymatic degradation by hyaluronidase, and injection force. While all tested fillers are suitable for volume restoration, BtHCROSS 2%^®^ demonstrates a distinctive combination of mechanical adaptability, structural support, enzymatic resistance, and low injection force, making it a versatile option for practitioners.

## 1. Introduction

Volume defines youth, symmetry, and gender traits. Deep fat loss hollows cheeks and deepens nasolabial folds [1]. Ageing, genetics, and lifestyle weaken ligaments, deplete collagen and elastin, and degrade muscle tone in the face, leading to wrinkles, sagging, deep creases, and a softened jawline, while external factors accelerate damage through oxidative stress [2]. Soft tissue fillers help counteract these changes, restoring volume and enhancing facial proportions [3]. Compared to permanent surgical interventions, biodegradable soft tissue fillers offer a safer, minimally invasive, and cost-effective alternative for facial rejuvenation.

Hyaluronic acid (HA) is the leading biodegradable filler in clinical use, favoured for its reliable safety and wide range of applications [4]. HA is a naturally occurring polysaccharide found in the skin, connective tissues, and joints, where it plays a crucial role in hydration and structural support [5]. HA fillers consist of HA gel, which can be crosslinked with variations in density and elasticity depending on the manufacturing process [6]. Their versatility allows for a broad range of applications, including facial contouring, wrinkle reduction, lip augmentation, and volume restoration [7]. Due to their biocompatibility and ability to be gradually reabsorbed by the body, HA fillers provide a temporary yet effective solution for aesthetic enhancement, making them a preferred choice for both practitioners and patients.

HA dermal fillers are modified using advanced crosslinking technologies to create injectable hydrogels with tuneable rheological properties. These include elasticity, viscosity, cohesivity, and durability [8]. These parameters are critical determinants of in vivo performance, influencing both the mechanical behaviour of the filler and its integration within the tissue.

Fillers intended for deep volumetric restoration typically exhibit a high degree of crosslinking, conferring increased viscosity and elasticity to provide structural support and resistance to displacement. Conversely, formulations designed for superficial applications, such as fine lines and perioral wrinkles, are generally less crosslinked—or not crosslinked at all—resulting in lower viscosity, enhanced spreadability, and softer tissue integration [9].

However, conventional rheological classifications often fail to capture the dynamic mechanical environment of the face, where fillers are subject to repetitive muscle activity and compressive forces. These biomechanical stresses affect not only the spatial distribution of the hydrogel but also its structural integrity and longevity. Therefore, achieving stable, natural-looking outcomes requires a balance between viscoelastic performance, adaptability to facial motion, and resistance to enzymatic degradation [10].

The clinical landscape of HA fillers continues to evolve, particularly in the domain of volume restoration. Since the introduction of Restylane^®^ (Galderma Laboratories), the first FDA-approved HA filler in 2003, newer-generation products have been developed with enhanced viscoelastic profiles tailored to deep tissue remodelling [11]. These formulations facilitate precise augmentation of anatomical regions such as the chin, jawline, midface, temples, and nasal dorsum.

Key performance attributes in these applications include high elasticity and cohesivity to maintain positional stability, alongside sufficient viscosity to enable controlled injection and accommodation of dynamic facial movement [12,13]. While the rheological profiles of many established fillers are well-characterised [13,14], their behaviour under cyclic mechanical stress—particularly in terms of elastic recovery and structural resilience—remains insufficiently studied. These characteristics are essential for ensuring long-term integration and aesthetic durability. Additionally, practical factors such as injectability and biodegradability remain important considerations for product selection in clinical practice.

This study compares five commercially available HA-based dermal fillers designed for volumisation: BtHCROSS 2%^®^, Juvederm Voluma^®^, Belotero Soft^®^, Restylane Refyne^®^, and Teosyal Ultimate^®^ (Table 1). These products share similar HA concentrations but differ in crosslinking technology, which is a critical determinant of rheology, cohesivity, degradation kinetics, and clinical performance.

Crosslinking technologies adopt distinct strategies to optimise these parameters. VYCROSS^®^ technology, used in Juvederm Voluma^®^, combines predominantly low-molecular-weight HA with a smaller fraction of high-molecular-weight HA to achieve efficient BDDE crosslinking, resulting in a dense, cohesive gel with reduced water uptake, high elasticity, and prolonged durability [15]. CPM^®^, the technology behind Belotero Soft^®^, creates a polydensified matrix with zones of varying crosslink density, producing a highly cohesive gel that integrates smoothly into superficial tissues [16]. XpresHAn^®^ (also known as OBT^®^), employed in Restylane Refyne^®^, modulates crosslinking density and HA concentration to enhance flexibility and adaptability, making it particularly suitable for dynamic facial areas [17]. RHA^®^, used in Teosyal^®^ Ultimate, applies a gentle, heat-free process that preserves long HA chains, yielding a resilient gel designed to mimic natural HA behaviour and maintain elasticity under facial motion [18]. In contrast, SARE^®^, the novel platform behind BtHCROSS 2%^®^, uses controlled-temperature synthesis and a tailored blend of high- and low-molecular-weight HA to form a homogeneous network with an optimised degree of modification and minimal residual BDDE. This design aims to balance structural support, adaptability, and injectability while ensuring high purity and safety. Technologies such as SARE^®^ have demonstrated enhanced resistance to degradation, tuneable rheological properties, and improved injectability. These transversal properties have led to successful applications in both aesthetic medicine and traumatology, offering superior performance and tailored solutions for clinical use [19].

These technological differences underscore the need for comparative physicochemical and rheological characterisation to link formulation design with potential clinical performance. The present study addresses this gap by evaluating the similarities and differences among these five fillers, focusing on chemical composition, structural stability, crosslinking degree, enzymatic degradation resistance, and injectability. Our findings highlight BtHCROSS 2%^®^ as a promising new filler designed to achieve volume enhancement with adaptability to facial motion.

**Table 1:** Commercial information on selected dermal fillers.

Commercial Sample	BtHCROSS 2%^®^	Juvederm Voluma^®^	Belotero Soft^®^	Restylane Refyne^®^	Teosyal^®^ Ultimate
Lot	1738/24	1000648978	B00041590	11512	23361BM1
HA mg/mL	20 mg/mL	20 mg/mL	20 mg/mL	20 mg/mL	22 mg/mL
Volume/Syringe	1 mL	1 mL	1 mL	1 mL	1 mL
Lidocaine	No	Yes	No	Yes	Yes
Crosslinking Technology	SARE^®^ [19]	VYCROSS^®^ [15]	CPM^®^ [16]	XpresHAn^®^ [17]	RHA^®^ [18]
Composition (IFU)	Sodium hyaluronate cross-linked 20 mg, disodium phosphate dihydrate 0.6 mg, sodium dihydrogen phosphate dihydrate 0.05 mg, sodium chloride 8 mg q.s. 1 mL	Hyaluronic acid gel 20 mg, lidocaine hydrochloride monohydrate 3 mg, phosphate buffer pH 7.2 q.s. 1 mL	Cross-linked sodium hyaluronate 20 mg, lidocaine hydrochloride 3 mg, phosphate buffer pH 7.0 q.s. 1 mL	Cross-linked hyaluronic acid 20 mg, Lidocaine hydrochloride, Phosphate-buffered saline pH 7.0 q.s. 1 mL	Crosslinked hyaluronic acid 22 mg, Lidocaine hydrochloride 3 mg, phosphate buffer pH 7.3 q.s. 1 mL

## 2. Results and Discussion

### 2.1. Chemical Composition and Degree of Modification

**Figure 1:**
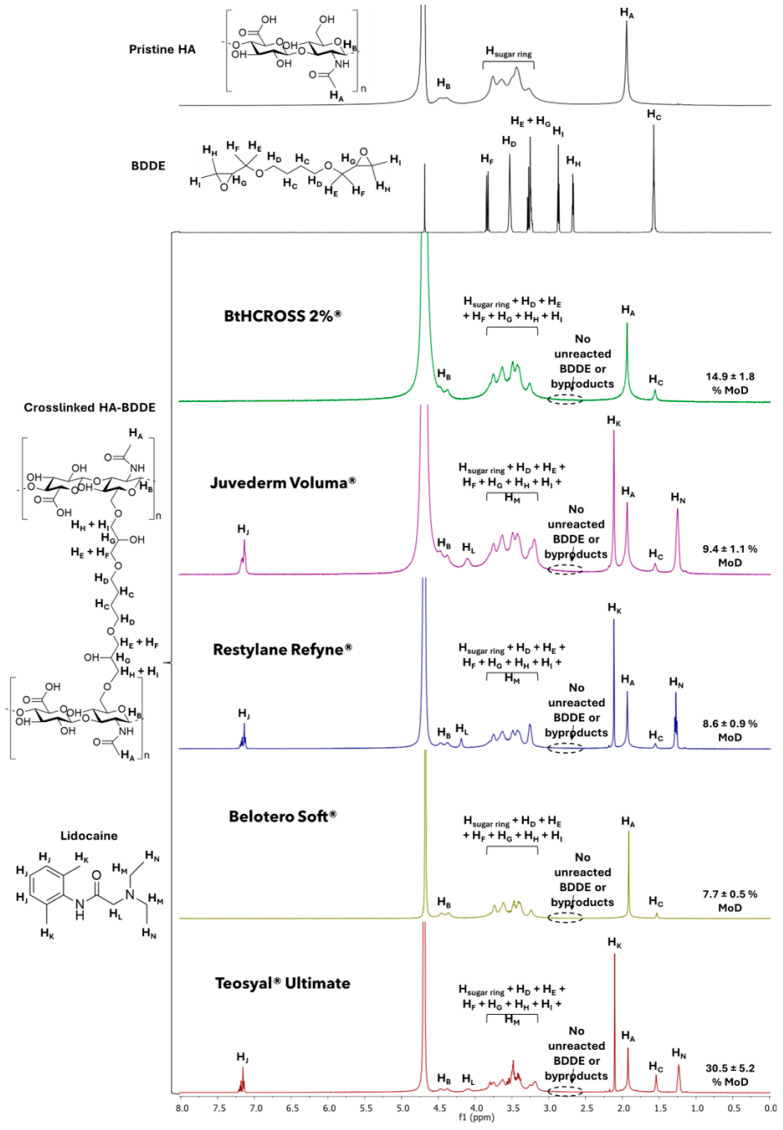
^1^H-NMR spectra of pristine HA, BDDE and commercial dermal fillers using D_2_O (4.70 ppm) as solvent and their MoD values.

We characterised the chemical composition of HA dermal fillers using proton nuclear magnetic resonance (^1^H-NMR) spectroscopy to determine the degree of modification (MoD) (Figure 1). Commercial HA hydrogels are typically crosslinked using 1,4-butanediol diglycidyl ether (BDDE), owing to its favourable biodegradability, biocompatibility, and chemical stability compared to alternative crosslinkers [20].

Initial ^1^H-NMR spectra were recorded for unmodified HA and BDDE to support peak identification and serve as reference profiles. The chemical shifts were validated against previously published data [21]. Distinct signals were observed at 1.90 ppm (methyl group of HA), 4.50 ppm (water), and within the 3.20–4.00 ppm range (protons of the sugar ring). BDDE displayed characteristic peaks at 1.60 ppm (H_C_), 2.70 ppm (H_H_), 2.85 ppm (H_I_), 3.30 ppm (H_E_ + H_G_), 3.60 ppm (H_D_), and 3.90 ppm (H_F_).

Crosslinked HA-BDDE hydrogels are formed via a base-catalysed ring-opening etherification reaction. In this process, hydroxyl groups on the HA backbone nucleophilically attack the epoxide moieties of BDDE, resulting in the formation of stable 1,4-butanediol di-(propan-2,3-diolyl) ether linkages between HA chains.

The ^1^H-NMR spectra obtained for BtHCROSS 2%^®^, Juvederm Voluma^®^, Belotero Soft^®^, Restylane Refyne^®^, and Teosyal^®^ Ultimate confirmed the formation of a crosslinked HA-BDDE network, as indicated by the presence of BDDE alkyl proton signals (H_C_) (Figure 1). Importantly, the absence of resonance peaks associated with unreacted BDDE and its byproducts in the 2.70–2.85 ppm region suggests successful hydrogel synthesis and efficient removal of residual BDDE (highlighted areas in Figure 1). This is a critical factor in ensuring the biocompatibility of medical devices, as unreacted monomers are known to be cytotoxic and pose serious health risks to patients [21,22].

^1^H-NMR analysis of crosslinked HA hydrogels provides quantitative information on their MoD, a parameter that influences gel hydration, mechanical properties, degradation kinetics, and in vivo performance as volumizing dermal fillers [23]. MoD was calculated by integrating the signal corresponding to the methylene protons of the crosslinker (H_C_, 1.60 ppm) relative to the methyl protons of the N-acetyl group (H_A_ 1.90 ppm) (Figure 1).

The results indicate that hydrogel Teosyal^®^ Ultimate exhibits the highest MoD at 30.5 ± 5.2%, followed by BtHCROSS 2%^®^ at 14.9 ± 1.8%. Hydrogels Juvederm Voluma^®^ (9.4 ± 1.1%), Restylane Refyne^®^ (8.6 ± 0.9%), and Belotero Soft^®^ (7.7 ± 0.5%) displayed lower MoD values. A higher MoD reflects a greater extent of chemical crosslinking, which is typically associated with increased gel stability and mechanical rigidity. Accordingly, hydrogel Teosyal^®^ Ultimate, with the highest MoD, is expected to possess high stiffness and resistance to degradation. Hydrogel BtHCROSS 2%^®^, with a moderate MoD, may offer a favourable balance between structural integrity and flexibility, making it potentially suitable for applications requiring both structural lift and adaptability in dynamic facial regions. In contrast, the lower MoD observed in Juvederm Voluma^®^, Belotero Soft^®^, and Restylane Refyne^®^ suggests softer, more malleable hydrogels with potentially greater hydration capacity and tissue integration.

These variations in MoD are consistent with previous data for Juvederm Voluma^®^ and Restylane Refyne^®^ [9,24], supporting the robustness of the present measurements, particularly for the intermediate crosslinking level observed in BtHCROSS 2%^®^. These findings suggest that Teosyal^®^ Ultimate is the least susceptible to hydration-induced volume expansion, with BtHCROSS 2%^®^ offering the second-best performance in this regard. Fillers BtHCROSS 2%^®^ and Teosyal^®^ Ultimate are therefore preferable for applications requiring precise volumisation with minimal unpredictability in swelling. To further understand the clinical implications of these compositional differences, a comparative rheological analysis was conducted to evaluate the mechanical performance of the hydrogels.

Fourier-transform infrared spectroscopy (FTIR) was employed to confirm the physicochemical structure of dermal fillers by analysing the characteristic vibration bands of HA, BDDE, lidocaine, and their combinations (Supplementary Figure S1).

The spectrum of pure HA (Supplementary Figure S1C) exhibits typical absorption bands at ~3300 cm^−1^ (O–H stretching), ~2900 cm^−1^ (C–H stretching), 1600–1650 cm^−1^ (carboxylate C=O stretching), and 1000–1150 cm^−1^ (C–O–C stretching). BDDE (Supplementary Figure S1A) shows distinctive fingerprint region bands at 910–950 cm^−1^ and strong C–O stretching between 1100 and 1200 cm^−1^, associated with epoxy groups. In contrast, lidocaine (Supplementary Figure S1B) presents characteristic amide and aromatic absorptions: N–H stretching near 3300 cm^−1^, aromatic C–H stretching around 3050 cm^−1^, C=O amide stretching at ~1650 cm^−1^, and aromatic skeletal vibrations below 1600 cm^−1^.

For dermal fillers, BtHCROSS 2%^®^ (and equivalently Belotero Soft^®^) displays spectral features of the HA–BDDE polymeric network (Supplementary Figure S1D). HA bands remain visible, but increased intensities at 1050–1150 cm^−1^ (C–O–C and C–O stretching) and 2800–2950 cm^−1^ (C–H stretching) confirm HA etherification with BDDE and the formation of stable ether bonds. The disappearance of the epoxy band near 910–950 cm^−1^ further indicates consumption of reactive BDDE groups in the crosslinked hydrogel.

In Juvederm Voluma^®^ (representative of Restylane Refyne^®^ and Teosyal^®^ Ultimate), the spectra combine HA–BDDE features with additional bands attributable to lidocaine (Supplementary Figure S1E). Amide I and II bands appear around 1650–1550 cm^−1^, and aromatic vibrations below 1500 cm^−1^ confirm the incorporation of the anaesthetic. These band assignments are consistent with previous FTIR-ATR analyses [21,25].

Thus, FTIR analysis effectively distinguishes the molecular components and structural modifications within dermal fillers. The presence and transformation of characteristic vibrational bands provide clear evidence of HA crosslinking with BDDE and the integration of lidocaine in specific formulations. These findings support the structural integrity and compositional accuracy of the analysed commercial products, reinforcing their intended functional properties in clinical applications.

### 2.2. Rheological Characterisation

HA fillers are viscoelastic materials that exhibit both viscous and elastic properties under shear deformation [26]. Upon injection, they are subjected to various mechanical forces, including shear stress from tissue movement, gravitational forces, and compression due to internal tissue pressure and external application. Understanding the rheological behaviour of HA fillers under mechanical stress is essential for predicting their clinical performance.

**Figure 2:**
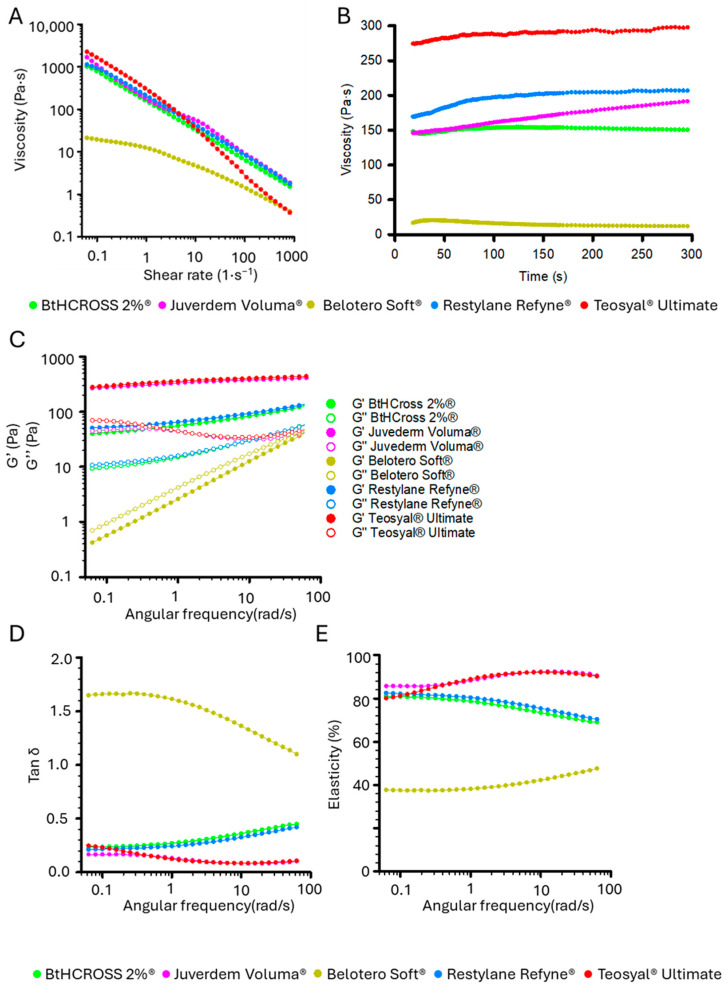
Rheological analysis: (**A**) Viscosity as a function of shear rate; (**B**) viscosity over time at a constant shear rate of 1 s^−1^; (**C**) storage modulus (G′) and loss modulus (G″) as functions of angular frequency; (**D**) loss factor (tan δ) versus angular frequency; and (**E**) elasticity (%) plotted against angular frequency.

Crosslinked HA hydrogels exhibit non-Newtonian behaviour, where viscosity decreases with increasing shear rate (shear thinning or pseudo-plasticity) [27], a property essential for smooth injectability. We measured the viscosity of various HA dermal fillers across shear rates from 0.05 s^−1^ to 1000 s^−1^. All fillers showed reduced viscosity with higher shear rates (Figure 2A), though formulations differed notably. BtHCROSS 2%^®^, Juvederm Voluma^®^, and Restylane Refyne^®^ had nearly identical profiles at both low and high shear rates. Belotero Soft^®^ displayed significantly lower viscosity, whereas Teosyal^®^ Ultimate had the highest viscosity at low shear rates (1663.92 Pa·s at 0.1 s^−1^), dropping sharply at higher rates (0.31 Pa·s at 10^3^ s^−1^), eventually converging with Belotero Soft^®^ (0.35 Pa·s, Figure 2A). Table 2 summarises these variations via viscosity shear ratios (η:γ): BtHCROSS 2%^®^ (109.54), Juvederm Voluma^®^ (100.44), and Restylane Refyne^®^ (131.52) versus Teosyal^®^ Ultimate (849.29) and Belotero Soft^®^ (33.74).

We further assessed the mechanical properties of all dermal fillers by monitoring viscosity at a constant shear rate of 1 s^−1^ over time (Figure 2B), providing insights into internal structure and stability. BtHCROSS 2%^®^, Juvederm Voluma^®^, and Restylane Refyne^®^ maintained viscosities of 150–200 Pa·s, indicating moderate crosslinking and stable networks suitable for consistent mechanical performance. Belotero Soft^®^ showed the lowest viscosity (10 Pa·s), suggesting a less dense network, whereas Teosyal^®^ Ultimate exhibited the highest (300 Pa·s), reflecting a highly crosslinked, robust structure. These viscosity trends correlate with crosslinking degree (MoD) determined by ^1^H-NMR (Section 4.2.1). All hydrogels remained stable over 300 s, with no signs of degradation or phase separation.

Further rheological evaluation was performed by analysing the storage modulus (G′) and loss modulus (G″) as functions of angular frequency (Figure 2C). Fillers Juvederm Voluma^®^ and Teosyal^®^ Ultimate exhibited the highest G’ values (378.00 Pa and 404.86 Pa at 10 rad/s, respectively), indicative of a predominantly elastic, solid-like structure with a high crosslinking density. Their elevated G’’ values (31.38 Pa and 34.65 Pa at 10 rad/s, respectively) reflect substantial energy dissipation, and capabilities to recover original shapes after an external stress (i.e., face motion) is removed.

In contrast, fillers BtHCROSS 2%^®^ and Restylane Refyne^®^ demonstrated lower G’ values (83.49 Pa and 93.06 Pa at 10 rad/s, respectively), suggesting a less rigid, still elastic matrix (Figure 2C). Although their G’’ values were initially lower, they increased significantly with frequency and converged with those of Juvederm Voluma^®^ and Teosyal^®^ Ultimate at higher angular velocities (30–58 Pa from 1 to 100 rad/s). This frequency-dependent behaviour implies a capacity to adapt under dynamic mechanical stress, making BtHCROSS 2%^®^ and Restylane Refyne^®^ potentially suitable for regions of the face subjected to frequent motion, where a balance between support and flexibility is required.

Filler Belotero Soft^®^, in contrast, exhibited a linear increase in both G′ and G″ across the tested frequency range, with G″ consistently exceeding G′ (Figure 2C). This viscoelastic profile indicates a predominantly viscous and weakly crosslinked network, typically associated with hydrogels designed for superficial soft tissue augmentation and lubrication, rather than deep volumetric restoration [28].

Loss factor or tan δ (G″/G′) was analysed against angular frequency to assess cohesivity, solidity, and elasticity across different strain rates (Figure 2D). A low tan δ (<0.5) indicates a predominantly elastic material suitable for structural support, while a high tan δ (>1) suggests a more fluid-like material that integrates well but may be less stable [26]. High-elasticity fillers (tan δ < 0.5) are ideal for lifting and volumisation, while fillers with higher tan δ (0.5–1) suit applications requiring soft, natural movement (e.g., lips, superficial wrinkles). BtHCROSS 2%^®^ and Restylane Refyne^®^ showed moderate tan δ (0.21–0.43), indicating balanced viscoelasticity for both support and integration. Belotero Soft^®^ had the highest tan δ (1.6–1.09), reflecting a fluid-like nature, whereas Juvederm Voluma^®^ and Teosyal^®^ Ultimate exhibited the lowest values (0.26–0.11), indicating highly cohesive gels that resist mechanical stress with increasing stiffness. In contrast, BtHCROSS 2%^®^ and Restylane Refyne^®^ had a moderate increase in tan δ, which ensures good response to mechanical stress, minimising stiffness and achieving a natural aesthetic outcome.

Elasticity as a function of mechanical stress was also evaluated (Figure 2E). At low angular frequencies, all fillers except Belotero Soft^®^ exhibited similar elasticity. Filler Belotero Soft^®^, possibly due to its low crosslinking density and higher viscosity, had a low elasticity (38%), which increased slightly under mechanical stress (48%) but remained lower than the other formulations. The fillers Juvederm Voluma^®^ and Teosyal^®^ Ultimate exhibited increasing elasticity with mechanical stress (80% to 90%), thus increasing resistance to mechanical deformation, whereas BtHCROSS 2%^®^ and Restylane Refyne^®^ showed a slight decrease (80% to 70%), ensuring structural support while preventing excessive rigidity, suitable for dynamic facial areas (Figure 2E). The combination of moderate elasticity and stability makes BtHCROSS 2%^®^ and Restylane Refyne^®^ particularly suited for applications requiring both volumisation and adaptability.

**Figure 3:**
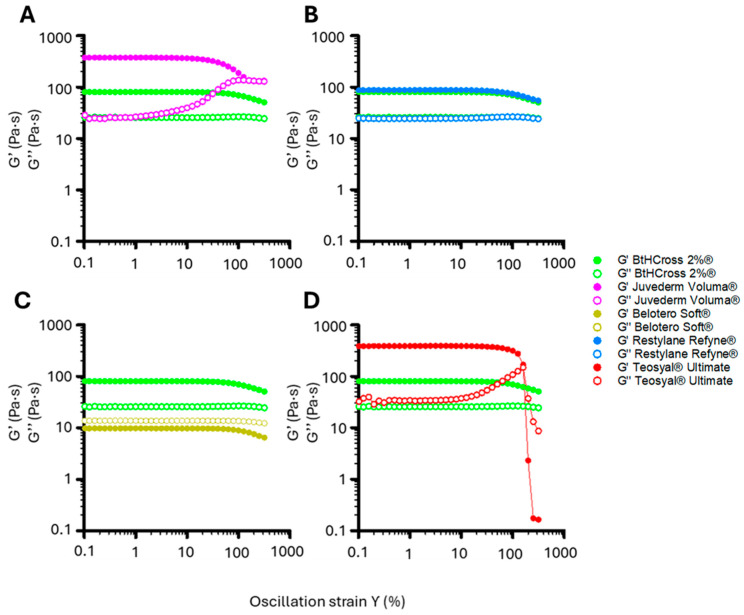
LVR represented as a function of G′ and G″ versus oscillation strain (%). BtHCROSS 2%® (green) is compared to (**A**) Juvederm Voluma®, (**B**) Restylane Refyne®, (**C**) Belotero Soft®, (**D**) Teosyal® Ultimate.

A highly crosslinked HA hydrogel with a high storage modulus (G′) may appear structurally robust, but prolonged mechanical stress can cause localised deformation, reduced integrity, and uneven degradation [29]. Conversely, a moderately crosslinked hydrogel with balanced elasticity and viscosity better absorbs and distributes stress, preventing contour irregularities and promoting smooth tissue integration [12]. Evaluating the linear viscoelastic regime (LVR) reveals how formulations respond to sustained loading, ensuring stability, adaptability, and longevity. Accordingly, we measured storage (G′) and loss (G″) moduli under increasing oscillatory strain to assess structural integrity under rising mechanical stress (Figure 3, Table 2).

BtHCROSS 2%^®^ maintained stable G′ and G″ up to 80% strain, then declined gradually to 300%, indicating strong structural integrity and elasticity across a wide strain range (Figure 3). Juvederm Voluma^®^ showed increased G″ beyond 10%, surpassing G′ at 100%, signalling a transition from solid- to fluid-like behaviour—strong for support but less ideal for dynamic regions (Figure 3A). Teosyal^®^ Ultimate, initially highly elastic, exhibited LVR collapse with sharp G’ decrease and G’’ rise beyond 100% strain (Figure 3D), suggesting network fracture under excessive deformation, suitable for rigid but not dynamic areas. Belotero Soft^®^ consistently displayed low G′ and G″, with G″ dominant at all strains, confirming its weakly crosslinked, viscous nature (Figure 3C). Restylane Refyne^®^ showed a similar LVR profile to BtHCROSS 2%^®^, confirming comparable mechanical stability for long-lasting, resilient applications (Figure 3B).

Rheological analysis of the five HA hydrogels highlights distinct viscoelastic behaviours that influence clinical performance (Figure 2 and Figure 3, Table 2). Effective fillers must balance structural support with adaptability to biomechanical forces for natural integration in dynamic facial regions.

Fillers Juvederm Voluma^®^ and Teosyal^®^ Ultimate exhibited the highest elastic (G′) and viscous (G″) moduli at low frequencies (0.1 rad/s), reflecting a dense, highly crosslinked network. However, under increasing mechanical stress, both showed a sharp increase in G″ and decline in tan δ, suggesting a transition to a more rigid, less adaptable state. This reduced mechanical compliance could hinder movement at the implantation site and compromise aesthetic outcomes.

Conversely, fillers BtHCROSS 2%^®^ and Restylane Refyne^®^ displayed more linear, stress-responsive viscoelastic profiles. Their G′ and G″ values increased gradually with frequency, indicating improved adaptability. This was supported by LVR analysis, which showed that both maintained structural integrity across the stress range tested, unlike Juvederm Voluma^®^ and Teosyal^®^ Ultimate, which exited the LVR prematurely. These properties suggest that BtHCROSS 2%^®^ and Restylane Refyne^®^ are better suited for applications requiring both structural support and mechanical flexibility.

Filler Belotero Soft^®^ exhibited distinct characteristics, with consistently higher G″ than G’ and a tan δ indicative of a predominantly viscous, fluid-like behaviour. Despite similar HA concentration and MoD to Juvederm Voluma^®^ and Restylane Refyne^®^, its rheological profile resembled that of a non-crosslinked filler, perhaps might be due to the presence of free HA chains. Nevertheless, these properties align with its commercial recommended use in fine-line correction and soft tissue augmentation.

In summary, filler BtHCROSS 2%^®^ offers a favourable balance of rigidity and adaptability, making it particularly well-suited for dynamic facial areas requiring natural movement and structural support [30].

### 2.3. Enzymatic Degradation Assay

Natural HA networks undergo continuous remodelling through the synthesis of new HA molecules and enzymatic degradation by hyaluronidase. Assessing the in vitro hydrolysis profile of HA-based hydrogels provides valuable insights into their potential in vivo behaviour, including their durability and structural integrity over time [31]. Understanding this information is essential for predicting the durability and clinical efficacy of HA-based dermal fillers.

**Figure 4:**
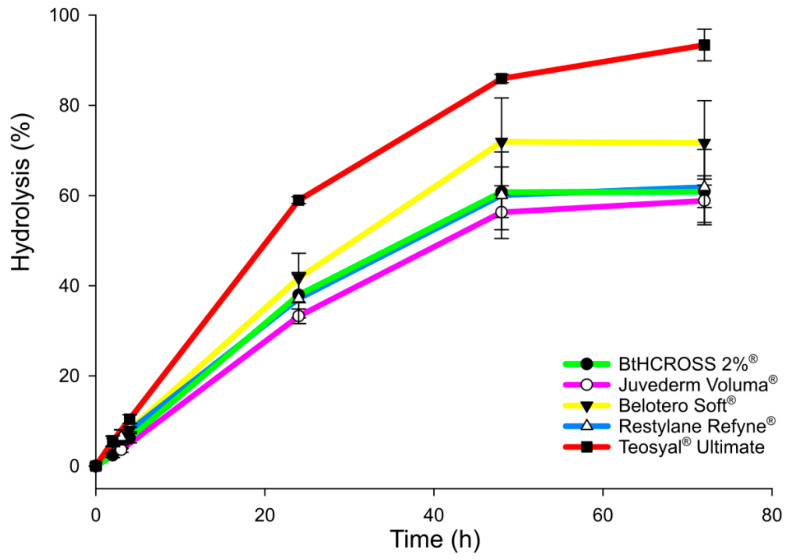
Enzymatic degradation profile of commercial dermal fillers. Released HA measured at 24 h, 48 h and 72 h.

Fillers BtHCROSS 2%^®^, Juvederm Voluma^®^, and Restylane Refyne^®^ showed similar enzymatic degradation profiles, each reaching approximately 60% hydrolysis within 48 h and stabilising by 72 h (Figure 4). This suggests moderate resistance to hyaluronidase under physiological conditions (PBS, 37 °C). In contrast, fillers Belotero Soft^®^ and Teosyal^®^ Ultimate degraded more rapidly, reaching 70–80% hydrolysis within 48 h (Figure 4). Notably, Teosyal^®^ Ultimate underwent near-complete degradation by 72 h, despite having the highest degree of modification (MoD) (Figure 1).

These findings indicate that MoD alone does not determine enzymatic stability. Factors such as particle size, HA molecular weight, and the presence of unbound or short-chain HA likely influence degradation kinetics [23]. Smaller particles would increase surface area for enzymatic attack, while low-molecular-weight or free HA chains could remain highly susceptible to hydrolysis. The rapid degradation of Teosyal^®^ Ultimate might reflect a higher proportion of such components, overriding the protective effect of crosslinking. In contrast, fillers such as BtHCROSS 2%^®^, Juvederm Voluma^®^, and Restylane Refyne^®^ exhibited the highest resistance to enzymatic breakdown, outperforming Belotero Soft^®^ and Teosyal^®^ Ultimate. This suggests that while crosslinking contributes to stability, it is the interplay of multiple formulation parameters—such as HA chain length and the ratio of crosslinked to free HA—that ultimately governs enzymatic resistance. Finally, BtHCROSS 2%^®^ demonstrated comparable stability to Juvederm Voluma^®^ and Restylane Refyne^®^, along with favourable rheological properties. This supports its suitability for clinical applications requiring durability and adaptability, and suggests it may exhibit similar behaviour in vivo.

### 2.4. Injectability

**Figure 5:**
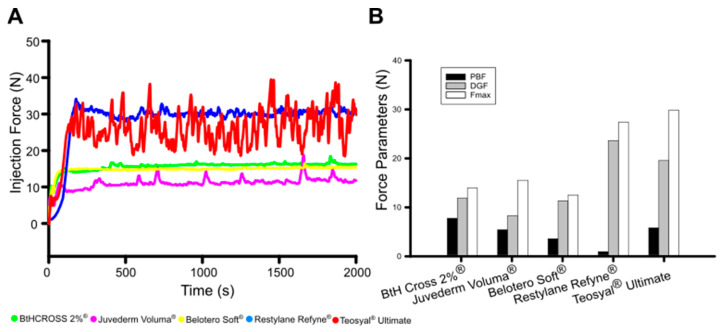
Injectability analysis: (**A**) Injection force (N) over time (s); (**B**) injectability force parameters including PBF (plunger-stopper break loose force or initial glide force), DGF (dynamic glide force), and Fmax (maximum force).

Subsequently, the injectability of crosslinked HA dermal fillers was assessed by monitoring the plunger-stopper break loose injection force over time (Figure 5A), including measurements of the initial glide force (PBF), peak force (Fmax), and dynamic glide force (DGF) (Figure 5B) [8]. The injection force data for the five crosslinked HA hydrogels revealed distinct differences in their rheological behaviour. Hydrogels BtHCROSS 2%^®^ and Belotero Soft^®^ exhibit a constant injection force of (DGF ≈ 15 N, Fmax < 20 N), indicating stable and predictable flow, which is desirable for smooth injection. Juvederm Voluma^®^ had a DGF of 10N but showed peaks (Fmax) at 15 N and 20 N, suggesting variability in injectability, potentially due to structural inhomogeneities or phase separation. In contrast, hydrogels Restylane Refyne^®^ and Teosyal^®^ Ultimate displayed a highly serrated profile, with a DGF ≈ 30 N with fluctuations between 27 N and 35 N (Fmax), indicating an unstable injection process, likely caused by gel heterogeneity, phase separation, or transient clogging effects.

Overall, the fluctuating forces in Juvederm Voluma^®^, Restylane Refyne^®^, and Teosyal^®^ Ultimate suggest inconsistencies that could lead to unpredictable injection experiences, poorer user-friendly injection performance, and patient discomfort. On the other hand, BtHCROSS 2%^®^ and Belotero Soft^®^ display a stable and moderate resistance, which makes them clinically most suitable from an injection performance point of view. Measurements of injection force confirmed that these two fillers provided the most stable and consistent injection profiles. Despite their differing viscoelastic and chemical properties, both exhibited favourable syringeability, with BtHCROSS 2%^®^ offering ease of injection without compromising on structural integrity. These properties underscore BtHCROSS 2%^®^’s suitability for a wide range of aesthetic indications, reinforcing its value in terms of both usability and patient comfort.

### 2.5. Linking Microstructure to Rheology, Injectability, and Degradation in Crosslinked HA Fillers

Microstructure plays a key role in HA filler performance, influencing tissue integration, mechanical resilience, and enzymatic stability. Even subtle differences in network architecture—such as density, cohesivity, and heterogeneity—can translate into clinically relevant variations. Understanding these features is therefore essential for predicting outcomes and guiding product selection.

Direct particle sizing of HA gels typically requires destructive steps (dilution, dispersion, drying) that disrupt cohesive networks or alter particulate interfaces. To preserve the hydrated state, we inferred structural characteristics from complementary, non-destructive measurements. Chemical composition was assessed by ^1^H-NMR (degree of modification, residual BDDE) and FTIR (etherification and anaesthetic-related bands). Rheological tests (G′, G″, tan δ, LVR) provided insight into network density and strain tolerance, while shear-rate sweeps revealed shear-thinning behaviour and potential domain disruption. Injectability force profiles reflected flow homogeneity, and enzymatic degradation assays indicated accessible surface area and the presence of free or short-chain HA.

BtHCROSS 2%^®^. Moderate MoD (~15%), mid-range G′, low–moderate tan δ, LVR > 300%; stable, low DGF; degradation comparable to Voluma^®^/Refyne^®^. Inference: Cohesive, resilient network with good homogeneity and limited brittle domains, enabling large-strain deformation and recovery. Smooth injectability supports uniform flow.

Restylane Refyne^®^. Similar rheology to BtHCROSS 2%^®^ but serrated injectability (higher DGF fluctuations). Inference: Adaptable network with good strain tolerance; serration suggests micro-heterogeneity (e.g., domains or phase inhomogeneities) affecting extrusion.

Juvederm Voluma^®^. High G′, low tan δ, early LVR exit (~180%), transient Fmax peaks; strong shear-thinning but stable DGF. Inference: Dense, cohesive matrix with limited strain tolerance—resists deformation at low strain but yields abruptly, consistent with tight crosslinking.

Teosyal^®^ Ultimate. Highest MoD (~30%), highest low-shear viscosity, very high shear-thinning ratio, early LVR exit (~219%), serrated injectability; faster degradation than MoD suggests. Inference: Stiff, densely crosslinked network with large domains prone to shear fragmentation and greater accessible surface area, explaining accelerated hydrolysis.

Belotero Soft^®^. G″ > G′ across frequency, highest tan δ, low viscosity at 1 s^−1^, stable low DGF; rapid degradation. Inference: Weakly crosslinked, highly hydrated matrix with abundant unbound/short HA chains, optimised for superficial spread rather than structural lift.

Overall, differences in rheology, injectability, and enzymatic stability among HA fillers can be linked to underlying network characteristics inferred from these complementary measurements. These findings highlight the limitations of oversimplified classifications, which fail to capture the complexity of current HA dermal fillers [30]. Modern technologies (e.g., SARE^®^, CPM^®^, VYCROSS^®^, XpresHAn^®^, RHA^®^) often produce cohesive gels with polydensified or hybrid architectures rather than purely particulate systems, and even cohesive matrices may contain micro-domains that influence extrusion and degradation. Integrating chemical, rheological, and functional assessments provides a more realistic framework for anticipating clinical performance and guiding product selection.

### 2.6. Safety Considerations:Lidocaine-Free Dermal Filler and Biological Safety

A key factor in comparing crosslinked HA dermal fillers is the presence of additional formulations, such as lidocaine. Lidocaine, a local anaesthetic, helps minimising injection discomfort. However, its inclusion affects both the injection process and treatment outcomes. Among available fillers, BtHCROSS 2%^®^ and Belotero Soft^®^ do not contain lidocaine, while Juvederm Voluma^®^, Restylane Refyne^®^, and Teosyal^®^ Ultimate include 3% lidocaine. While this anaesthetic enhances patient comfort, using HA fillers without lidocaine offers distinct advantages, including increased safety and customization. Avoiding lidocaine reduces the risk of allergic reactions and localised side effects, such as redness, irritation, hives, rash, itching, bruising, and swelling. In severe cases, symptoms may include pale or bluish skin and headaches [32,33,34]. Moreover, accidental injection in vascular system may be unnoticed when using lidocaine [35]. Additionally, clinicians should be aware of potential drug interactions affecting lidocaine metabolism, as coadministration of lidocaine with paracetamol may prolong unwillingly lidocaine’s pharmacokinetics beyond its expected duration, increasing serum concentrations [36]. This interaction could influence dosing strategies, requiring careful consideration in clinical applications to prevent unintended prolongation of anaesthetic effects.

Some practitioners prefer to administer anaesthetics topically before dermal filler application for better dosage control, tailoring pain management to each patient’s needs. Additionally, as lidocaine is a vasodilator, it can cause temporary swelling and increased bruising. Fillers without lidocaine might result in more predictable aesthetic outcomes as research indicates that lidocaine increases injection force requirements [37], which might explain the fluctuating injection force profiles observed for those fillers with lidocaine 3% (Figure 5).

The biological safety of BtHCROSS 2%^®^ is further reinforced compared to other medical devices, as it not only meets the biocompatibility and endotoxin regulatory requirements for Class III medical devices (<0.5 EU/mL based on EP <2.6.14>, EP <5.1.4> and USP <85>) and in addition adheres to more stringent standards set for ophthalmic products (<0.2 EU/mL, ISO 15798:2022) [38]. This high level of purity ensures a reduced risk of inflammatory reactions and enhances the biocompatibility of the product, making it particularly suitable for medical and aesthetic applications. By complying with these rigorous safety thresholds, BtHCROSS 2%^®^ offers healthcare professionals and patients greater confidence in its quality, reliability, and overall performance.

## 3. Conclusions

This in vitro analysis highlights the clinical relevance of key physicochemical parameters—rheology, degree of modification, enzymatic stability, and injection force—in defining the performance of HA-based dermal fillers. Rheological properties (G′, G″, and LVR) determine structural support and adaptability, MoD influences volumetric predictability, enzymatic stability correlates with in vivo durability, and injection force impacts handling precision.

Juvederm Voluma^®^ and Teosyal^®^ Ultimate exhibited high stiffness and lifting capacity, favouring deep volumisation, but their early LVR exit and resistance to mechanical deformation may limit use in dynamic areas. Belotero Soft^®^, with its viscous-dominant profile aligns with superficial, fine-line correction where spreadability and reversibility are prioritised. Both Belotero Soft^®^ and Teosyal^®^ Ultimate also exhibited a faster degradation rate, thus likely limiting durability in vivo. Restylane Refyne^®^ demonstrated adaptability and durability through preserved LVR and high enzymatic resistance, though injection-force variability may affect consistency.

**Figure 6:**
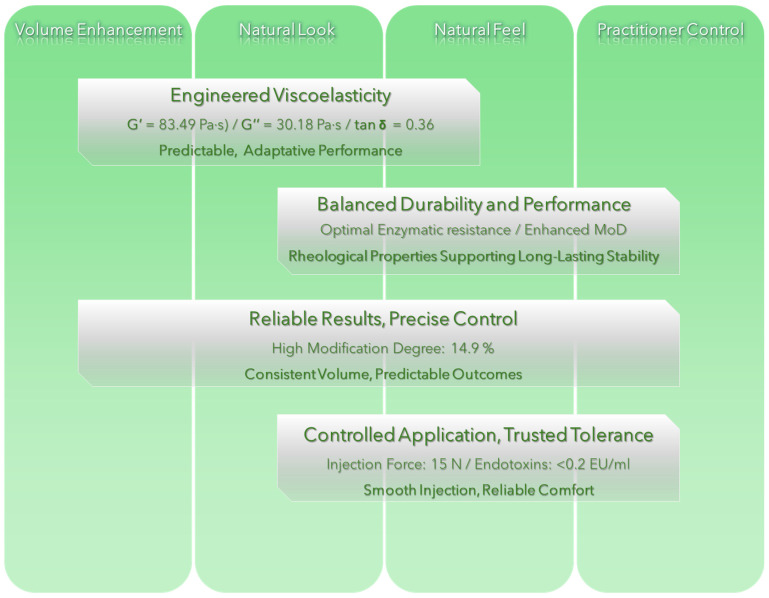
Overview of key biophysical and rheological features of BtHCROSS 2%^®^.

Among the evaluated fillers, BtHCROSS 2%^®^ emerges as a well-balanced option (Figure 6), encompassing a unique combination of physicochemical characteristics offering predictable viscoelastic behaviour, degradation, and optimal injectability. Its moderate MoD ensures precise volume enhancement which may reduce post-injection swelling, while its rheological properties allow for adaptable structural support without compromising natural facial dynamics, thereby supporting clinical usability and patient comfort. Furthermore, BtHCROSS 2%^®^ maintains stable injection force requirements, enhancing clinical ease of use. Thus, by balancing longevity, adaptability, and aesthetic outcomes, BtHCROSS 2%^®^ properties may facilitate precise volume restoration in various clinical contexts.

## 4. Materials and Methods

### 4.1. Materials and Chemicals

This study compared five commercially available injectable hydrogels composed of chemically crosslinked HA, designed for use as dermal fillers: BtHCROSS 2%^®^ (i+Med S. Coop., Vitoria-Gasteiz, Spain), Juvederm Voluma^®^ (Allergan Aesthetics, Irvine, CA, USA), Belotero Soft^®^ (Merz Pharma GmbH & Co., Frankfurt am Main, Germany), Restylane Refyne^®^ (Galderma Laboratories, L.P., Fort Worth, TX, USA), and Teosyal^®^ Ultimate (Teoxane Laboratories, Geneva, Switzerland). Their specifications are outlined in Table 1.

Proton nuclear magnetic resonance spectroscopy (^1^H-NMR) was performed using deuterium oxide (D_2_O, 99.8 atom % D, Across Organics, (Antwerpen, Belgium)). To prepare phosphate-buffered saline (PBS, pH 7.4), the following reagents were obtained from Fischer Chemicals (Zurich, Switzerland): disodium hydrogen phosphate dodecahydrate (Na_2_HPO_4_·12H_2_O, 99%), sodium dihydrogen phosphate dihydrate (NaH_2_PO_4_·2H_2_O, 99%), and sodium chloride (NaCl, 99%). Spectra/Por membranes (12–14 kDa) were supplied by Iberlabo (Madrid, Spain), and hyaluronidase (derived from bovine testes, 400–1000 units/mg solid) was sourced from Sigma-Aldrich (St. Louis, MO, USA).

Commercial HA-based dermal fillers are classified as Class III medical devices, as they are considered implants that remain in contact with skin, tissue, or bone for periods exceeding 30 days. Given this classification, these injectable hydrogel devices fall under the highest risk category. Consequently, in addition to physicochemical characterisation, further testing was conducted to ensure compliance with regulatory standards.

### 4.2. Characterisation

#### 4.2.1. Chemical Composition and Degree of Modification (MoD)

Chemical composition of HA-based commercial hydrogels was investigated by proton nuclear magnetic resonance spectroscopy (^1^H-NMR) using Bruker AVANCE III NMR spectrometer (500 MHz) (Bruker BioSpin, Rheinstetten, Germany). ^1^H-NMR spectra were obtained in deuterated water (30 mg/mL concentration). MoD of hydrogels was calculated from the ratio between the integrals of the following signals (Equation (1)).

(1) $$Degree\;of\;Modification\left(MoD\right)=(I^{\delta H1.60}/4)/(I^{\delta H1.90}/3)\cdot 100$$

where *I^δH^*^1.60^ and *I^δH^*^1.90^ are the integrations of protons at 1.60 ppm and the methyl protons of the *N*-acetyl groups at 1.90 ppm, respectively.

#### 4.2.2. Fourier Transform Infrared Spectroscopy with Attenuated Total Reflectance Methodology (FTIR-ATR)

A FTIR spectrometer (Nicolet Nexus, Thermo Scientific, Loughborough, UK) was employed using ATR sampling technique to determine the infrared spectra of pure HA, BDDE, Lidocaine, HA-BDDE and HA-BDDE-Lidocaine samples. Spectra were recorded in the 400–4000 cm^−1^ range at a 4 cm^−1^ resolution and 32 scans/spectrum.

#### 4.2.3. In Vitro Enzymatic Degradation

A total of 0.5 g of each commercial HA-based hydrogel was placed inside a dialysis membrane containing 5 mL of hyaluronidase solution in PBS (200 IU/mL, pH 7.4). The samples were then incubated at 37 °C for 72 h in 20 mL of the same enzyme solution. At predetermined time points, 0.5 mL aliquots were collected and stored at −20 °C until analysis. The extent of HA degradation was quantified using a Spectramax PC340 UV-VIS spectrophotometer (Molecular Devices, San Jose, CA, USA) via the carbazole assay [39], with absorbance measured at 530 nm.

#### 4.2.4. Rheological Characterisation

Rheological properties were analysed using an AR550 rheometer (TA Instruments, New Castle, DE, USA) equipped with a standard 40 mm diameter steel cone-plate geometry. The testing protocol included shear rate sweep, time sweep under constant shear, and oscillatory frequency sweep. Viscosity (η) was determined during the time sweep at a constant shear rate of 1 s^−1^. For the shear rate sweep, the shear rate (γ) was varied from 0.1 to 1000 s^−1^. Subsequently, the viscosity-to-shear rate ratio was calculated according to Equation (2):

(2) $$\eta :\gamma \;ratio=\frac{{\eta \;at\;\gamma \;1\;s}^{-1}\;}{{\eta \;at\;\gamma \;1000\;s}^{-1}}$$

Additionally, the elastic modulus (G′) and the viscous modulus (G″) were determined as functions of frequency ranging from 0.1 to 100 Hz, with the strain fixed at 1%. Based on these measurements, the complex modulus (G), loss tangent (tan δ), and percentage elasticity were subsequently calculated using the corresponding equations.

(3) $$Complex\;modulus\;(G^{*})=\sqrt{{\left(G\prime \right)}^{2}+{\left(G\prime \prime \right)}^{2}}$$

(4) $$Loss\;factor\;(\tan\delta )=\frac{G\prime \prime }{G\prime }$$

(5) $$Elasticity\;\left(\%\right)=\frac{G\prime }{G\prime +G\prime \prime }\cdot 100$$

The linear viscoelastic regime (LVR) was assessed by an oscillatory strain sweep, carried out at a frequency of 1.0 Hz and a strain ranging from 0.1% to 300%.

#### 4.2.5. Injectability

To assess the injectability of the commercial hydrogels, the extrusion force was measured using a PCE/FB200 dynamometer (PCE Instruments, Jupiter, FL, USA) integrated with a SAUTER TVM 5000N230N (Kern & Sohn GmbH, Balingen, Germany) motorised vertical stand operating at a compression speed of 40 mm·min^−1^. The syringe and needle used for testing were those supplied by the respective manufacturer.

### 4.3. Statistical Analysis

Quantitative data are presented as mean ± standard deviation (SD), based on a minimum of *n* = 3 independent replicates per group, unless otherwise indicated. Statistical analyses were conducted using Microsoft Excel and GraphPad Prism 10.0, and graphical representations using SigmaPlot 15.0. Differences between groups were assessed using two-tailed *t*-tests. A *p*-value ≤ 0.05 was considered statistically significant.

## Figures and Tables

**Table 2:** Rheological analysis of the dermal fillers under study. Values for G′ and G″ were obtained at an angular frequency of 10 rad/s. For the linear viscoelastic region (LVR), the reported strain values (γ%) correspond to the point where the storage modulus (G′) intersects with the loss modulus (G″). ‘N.A.’ indicates that G″ remained dominant over G′ throughout the measurement, indicating a primarily viscous response.

Commercial Sample	BtHCROSS 2%^®^	Juvederm Voluma^®^	Belotero Soft^®^	Restylane Refyne^®^	Teosyal Ultimate^®^
η at γ 1 s^−1^ (Pa·s)	143.65	159.70	11.81	192.02	263.28
η at γ 1000 s^−1^ (Pa·s)	1.31	1.59	0.35	1.46	0.31
η:γ ratio	109.54	100.44	33.74	131.52	849.29
G′ (Pa)	83.49	378.22	12.63	93.06	404.86
G″ (Pa)	30.18	31.38	17.24	30.34	34.65
tan δ	0.36	0.08	1.37	0.33	0.09
Elasticity (%)	73.45	92.34	42.28	75.41	92.12
LVR (γ%)	>300	181.20	N.A.	>300	218.77

## Data Availability

All data generated or analysed during this study are included in this published article.

## Supplementary Materials

The following supporting information can be downloaded at: https://www.mdpi.com/article/10.3390/gels11090754/s1, Figure S1: FTIR analysis of dermal filler formulations.

## Abbreviations

The following abbreviations are used in this manuscript:

BDDE	1,4-butanediol diglycidyl ether
FTIR	Fourier Transform Infrared Spectroscopy
HA	Hyaluronic Acid
^1^H-NMR	Proton Nuclear Magnetic Resonance
LVR	Linear Viscoelastic Regime
MoD	Degree of chemical modification
