# Supplementary figures and images for: Viscoelastic Properties and Enzymatic Degradation of Crosslinked Hyaluronic Acid for Deep Dermal Filler Use
Source: Gels. 2025 Sep 18;11(9):754. doi: 10.3390/gels11090754 (PMC12469832; doi:10.3390/gels11090754)

Supplementary Figure 1

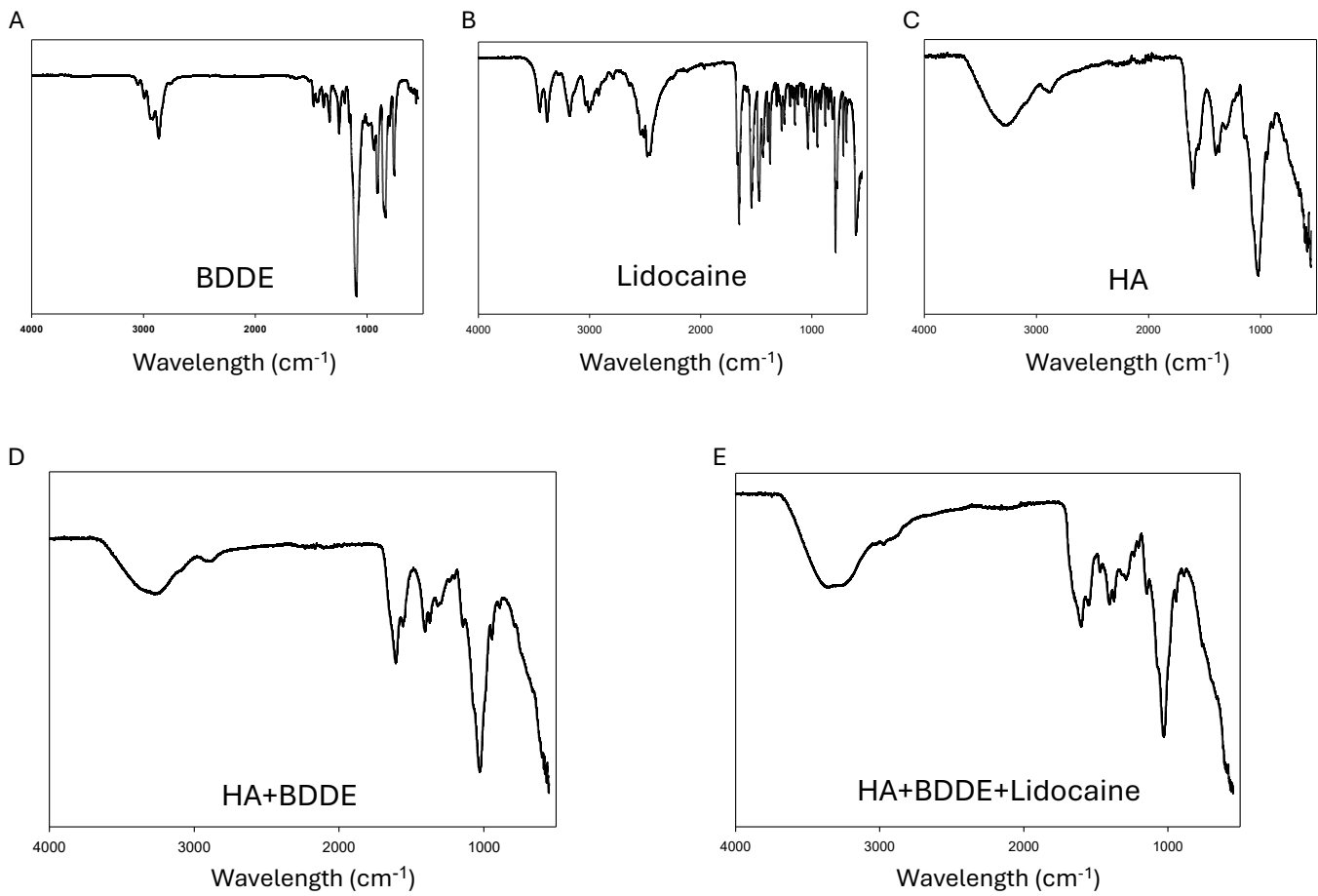

Supplement: Supplementary file 1 [file gels-11-00754-s001.zip › gels-3821909-supplementary.pdf]
